# Overlapping syndrome of anti-MOG antibody-associated disease and anti-mGluR5 encephalitis manifested as optic neuritis: A case report

**DOI:** 10.1097/MD.0000000000039146

**Published:** 2024-08-16

**Authors:** Jianhang He, Xiaoyan Niu, Xiaoyan Chen, Boya Ma, Yazhou Ren, Weimin Qi, Xiuping Zhan, Yue Meng, Jianxia Li, Haining Li

**Affiliations:** a Department of Neurology, General Hospital of Ningxia Medical University, Yinchuan, China.

**Keywords:** encephalitis, myelin oligodendrocyte glycoprotein, metabotropic glutamate receptor 5, overlapping syndrome

## Abstract

**Rationale:**

Anti-Myelin oligodendrocyte glycoprotein (MOG) and anti-metabotropic glutamate receptor 5 (mGluR5) double antibody positive encephalitis characterized by optic neuritis is extremely rare. We present a case of overlapping syndrome of MOG-IgG-associated disease and anti-mGluR5 encephalitis manifested as optic neuritis.

**Patient concerns:**

A 60-year-old Chinses woman presented to the hospital with progressive vision loss and headache for 1 week. The cerebrospinal fluid examination was within the normal range. Visual evoked potentials study disclosed prolonged latency of P100 bilaterally. Fundus examination revealed indistinct boundaries of both optic discs. Her brain magnetic resonance imaging showed patchy hyperintensity in the posterior horn of the left ventricle and the left optic nerve. Her serum was positive for anti-MOG and anti-mGluR5 antibodies.

**Diagnosis:**

The patient was diagnosed with overlapping syndrome of anti-MOG antibody-associated disease and anti-mGluR5 encephalitis mainly based on the clinical symptoms and further test of the antibody in serum.

**Interventions and outcomes:**

She was subsequently subjected to empirical treatment with intravenous methylprednisolone. After discharge, she was given a tapering dose of oral prednisone, alongside mycophenolate mofetil. On outpatient follow-up, her symptoms showed no relapse after 1 month, and her condition remained stable.

**Lessons:**

Early recognition of autoimmune encephalitis is crucial. The detection of cerebrospinal fluid and serum of autoimmune encephalitis and demyelinating diseases of the CNS, including MOG-IgG and mGluR5-IgG, should be strengthened in order to make a precise diagnosis and develop a comprehensive treatment plan in a timely manner.

## 1. Introduction

Myelin oligodendrocyte glycoprotein (MOG) is a myelin glycoprotein specifically expressed in the oligodendrocytes of the central nervous system.^[1]^ Degradation and demyelination of oligodendrocytes is the result of activation of the complement cascade by anti-MOG antibodies.^[2]^ MOG-associated disease (MOGAD), a novel autoimmune disorder, primarily presents itself as central nervous system (CNS) demyelination. The primary clinical manifestations include optic neuritis, transverse myelitis, brainstem demyelination, and acute disseminated encephalomyelitis.^[3]^ Metabotropic glutamate receptor 5 (mGluR5) is a type of G-protein coupled receptors that is activated by binding to the excitatory neurotransmitter glutamate. Anti-mGluR5 encephalitis is an unusual and complex autoimmune disorder that affects the nervous system. To date, only 20 cases have been reported worldwide. Anti-mGluR5 encephalitis is characterized by cognitive impairment, psychiatric disturbances, seizures, disturbance of consciousness, and sleep disorders. Notably, the literature suggests mGluR5’s role in guiding the differentiation of oligodendrocyte precursors into mature oligodendrocytes, which may elucidate the concurrent incidence of mGluR5 and MOG.^[4]^ So far, MOG and mGluR5 double antibody positive encephalitis characterized by optic neuritis is extremely rare. Here, we present a case of overlapping syndrome of MOG-IgG-associated disease and anti-mGluR5 encephalitis manifested as optic neuritis. The purpose of this study is to enrich the extant literature and provide a reference for the clinical diagnosis and treatment of overlapping syndromes.

## 2. Case report

**Figure 1.**
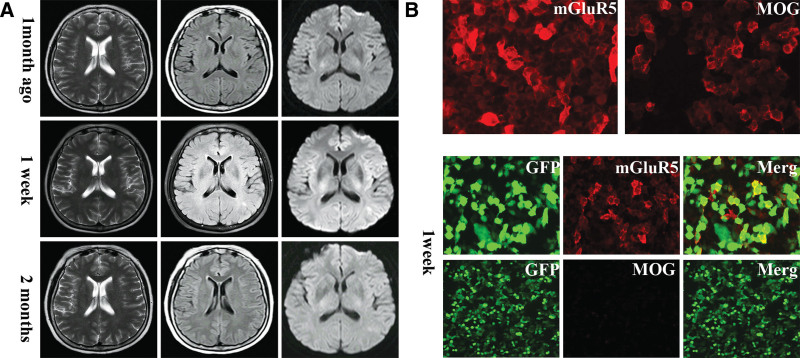
(A) Brain magnetic resonance imaging (MRI) of the patient at 1 month before the symptom onset was normal. Brain MRI of the patient at 1 week after the symptom onset reveled abnormal signals in the posterior horn of the left lateral ventricle, with T2-weighted, T2-FLAIR, and DWI sequence of hyperintensity. Brain MRI repeated at 2 months and performed a significant improvement of the imaging abnormality. (B) Immunofluorescence of anti-mGluR5 and MOG antibodies in the patient’s cerebrospinal fluid and serum. The detection principle is realized by cell-based assay (CBA). Fluorescent antibody staining for expression of mGluR5 and MOG antibodies in the serum of the patient at 1 week after the symptom onset. Fluorescent antibody staining for expression of mGluR5 and MOG antibodies in the serum of the patient after being released from the hospital. DWI = diffusion weighted imaging, FLAIR = fluid attenuated inversion recovery, mGluR5 = metabotropic glutamate receptor 5, MOG = myelin oligodendrocyte glycoprotein.

A 60-year-old Chinses woman presented to the hospital with progressive vision loss and headache for 1 week. She has a history of sleep disorders for 1 year and has been given zolpidem tartrate for a long time to relieve her symptoms. No history of infection or vaccination prior to symptom onset was reported. Neurological examination revealed that except for decreased vision in both eyes, other aspects were not obvious. Visual acuity assessments indicated 0.4 vision in the right eye and only manual visible in the other eye. Fundus test revealed indistinct boundaries of both optic discs. Laboratory workup showed routine biochemical tests, antinuclear antibody, hepatitis antibody, syphilis antibody, and HIV antibody were normal. Malignancy workups, incorporating peripheral blood tumor markers, chest CT scans, and abdominal color ultrasounds, returned negative. A brain magnetic resonance imaging (MRI) performed 1 month ago showed no obvious abnormalities (Fig. 1A). However, a subsequent MRI upon admission showed patchy hyperintensity in the posterior horn of the left lateral ventricle on T2-weighted, T2-fluid attenuated inversion recovery imaging and diffusion weighted imaging (Fig. 1A). Besides, her brain MRI showed patchy hyperintensity before the optic chiasm in the left optic nerve on T2-FLAIR imaging. Her spinal cord MRI appeared normal. Visual evoked potentials study disclosed prolonged latency of P100 bilaterally. No abnormalities were detected in the electroencephalogram. We suspected that she may have demyelination-related disease. Thus, a lumbar puncture was performed 1 day later, demonstrating an intracranial pressure of 140 mmH_2_O. Cerebrospinal fluid (CSF) examination was in the normal range, including leukocytes at 1/mm^3^, glucose at 3.9 mmol/L, chlorides at 127 mmol/L, and protein at 0.23 g/L. The CSF alcian blue staining, bacterial and fungal cultures were negative. Concurrently, CSF and serum tests for MOG antibody and an autoimmune encephalitis panel covering antibodies against NMDA, AMPA1, AMPA2, LGI1, CASPR2, GABAB, GABAA, IgLON5, DPPX, GlyR1, DRD2, GAD65, mGluR5, mGluR1, KLHL11, AQP4, MOG, GFAP, and neurexin-3α were conducted on a cell-based assay. The autoimmune encephalitis panel indicated positivity for serum MOG antibodies (1:10) and serum mGluR5 antibody (1:32), while a negative result was observed in CSF (Fig. 1B). This led to a diagnosis of overlapping syndrome involving MOG antibody-associated encephalitis and mGluR5 antibody autoimmune encephalitis.

The patient was subsequently subjected to empirical treatment with intravenous methylprednisolone involving a step-down dosage regimen over 12 days. On the seventh day of treatment, her headache and loss of vision were relieved. After discharge, she was given a tapering dose of oral prednisone (60 mg/d), alongside mycophenolate mofetil (1000 mg/d). On outpatient follow-up, her symptoms showed no relapse after 1 month, and her condition remained stable. Reexamination of MOG antibody and mGluR5 antibody revealed serum MOG antibody negativity and a decrease in serum mGluR5 antibody titer to 1:10 (Fig. 1B). The follow-up fundus examination was normal and visual acuity assessments indicated 0.6 vision in both eyes. The repeated brain MRI showed significant regression of the lesions in the posterior horn of the left lateral ventricle and no new lesions (Fig. 1A).

## 3. Discussion

This study described an unusual instance of overlapping syndrome, characterized by the simultaneous occurrence of MOG and mGluR5 antibodies manifested as optic neuritis. Given their exceptional rarity, the underlying mechanisms driving their coexistence remain elusive. Notably, mGluR5 has been identified as playing an important role in the differentiation process from oligodendrocyte precursor cells to oligodendrocytes, suggesting a potent association of this antibody with myelin dysfunction^.[4]^ MOG has also been implicated in the formation and maintenance of the myelin sheath. Thus, we hypothesize that the reduction of mGluR5-associated oligodendrocytes may be accompanied by changed MOG signals. This could provide evidence for the coexistence of mGluR5-IgG and MOG-IgG.

Despite the MOG-IgG titer presenting at a minimally elevated level in our case, its significance remains substantial when integrated with supplementary clinical or MRI indicators. She had typical clinical manifestations of optic neuritis, supported by fundus examination, VEP and brain MRI. Therefore, the diagnosis of MOGAD in this patient was reasonable.

MOGAD is a CNS demyelinating disorder, typically observed to follow a monophasic or relapsing trajectory of neurological dysfunction.^[5]^ Generally, steroid treatment is almost always effective for patients afflicted with MOGAD. Optic neuritis is the most common clinical manifestation of MOGAD, succeeded by myelitis and acute disseminated encephalomyelitis. Prior investigations attest that visual impairment in MOGAD patients during the acute phase tends to be more profound, but that the vision of the majority of patients improves considerably with consistent medical intervention.^[6]^ In this case, her brain MRI revealed a patchy focus in the posterior horn of the left lateral ventricle. Currently, it remains challenging to distinguish if the lesion is induced by MOGAD, anti-mGluR5 encephalitis, or both, given either type of encephalitis could be associated with such presentation. We consider that the focus is mainly related to demyelination.

The incidence of anti-mGluR5 encephalitis in clinical settings is remarkably low, mainly manifesting cognitive deficit, mental disorders, epilepsy, disturbance of consciousness, and sleep disorders. Anti-mGluR5 encephalitis has been reported more frequently as the rapid advancements in detection techniques for encephalitis-associated antibodies. The mGluR5 antibody was initially detected in 2011 serum samples from limbic encephalitis and Hodgkin’s lymphoma patients.^[7]^ Furthermore, it has been reported that over half of the patients with anti-mGluR5 encephalitis in Western countries exhibit a correlation with tumors (primarily Hodgkin’s disease). However, in China, only 13% of patients with anti-mGluR5 encephalitis have related tumors.^[8]^ Patients with anti-GluR5 encephalitis typically exhibited a favorable response to immunotherapy, and the majority of them had recovered completely or partially at their latest follow-ups.^[8,9]^ Despite the fact that anti-mGluR5 encephalitis was categorized as limbic encephalitis, brain MRI scans have revealed the potential involvement of extra-limbic lesions, either independently or in conjunction with limbic lesions. In the event of suspected disease recurrence, repeated brain MRI is necessary. In this case, she was initially diagnosed with MOGAD and treated with steroids. However, the CSF/serum autoantibody test results forced us to reconsider the diagnosis, even if she had no obvious clinical symptoms associated with anti-mGluR5 encephalitis. Recently, a case series and literature review of mGluR5 antibody encephalitis from China showed that mGluR5 encephalitis in combination with other antibody positivity is often characterized by atypical clinical manifestations.^[8]^ Although our patient is also complicated with MOG antibody positive encephalitis, the difference is that she presents with typical optic neuritis. Thus, it can be seen that the clinical manifestations of mGluR5 encephalitis in this case are not prominent and seem to be overshadowed by the clinical manifestations of other antibody-positive encephalitis. However, the current limit on the number of cases, the underlying mechanism of this phenomenon is not clear and needs to be further studied.

Although MOG and mGluR5 antibodies coexist, the initial treatment for patients with both antibodies converges with that for individuals harboring a single positive antibody. Patients with MOGAD seemed to respond well to steroid therapy, whether they are part of the overlapping syndrome or not.^[10]^ A higher recurrence risk is reported in patients manifesting overlapping symptoms.^[11,12]^ In our case, we reexamined the serum MOG antibody and mGluR5 antibody, in which the MOG antibody was negative and the mGluR5 antibody titer decreased to 1:10. Given the persistent positivity to serum mGluR5-IgG, it is of utmost importance to maintain consistent immunomodulatory treatment and perform regular antibody titer detection during follow-up. Moreover, a minority of patients with anti-mGluR5 encephalitis are also associated with neoplasms,^[8]^ particularly in patients from the West. Given that tumors themselves may be insidious and some patients are diagnosed years after recovery from autoimmune encephalitis, regular tumor screening is of great importance.

## 4. Conclusion

We describe an exceptionally unusual case of overlapping syndrome involving MOGAD and anti-mGluR5 encephalitis. For patients with unexplained encephalitis, the overlapping syndrome of different antibody-associated encephalitis should also be considered. The detection of cerebrospinal fluid and serum of autoimmune encephalitis and demyelinating diseases of the CNS, including MOG-IgG and mGluR5-IgG, should be strengthened in order to make a precise diagnosis and develop a comprehensive treatment plan in a timely manner.

## Author contributions

**Conceptualization:** Haining Li.

**Data curation:** Xiaoyan Niu.

**Project administration:** Boya Ma, Xiaoyan Chen, Weimin Qi, Xiuping Zhan, Yue Meng, Jianxia Li.

**Resources:** Jianhang He.

**Supervision:** Haining Li.

**Writing – original draft:** Jianhang He.

**Writing – review & editing:** Xiaoyan Niu, Yazhou Ren.
